# Therapeutic efficacy of macrolides in management of patients with mild COVID-19

**DOI:** 10.1038/s41598-021-95900-z

**Published:** 2021-08-11

**Authors:** Alaa Rashad, Asmaa Nafady, Mohammed H. Hassan, Haggagy Mansour, Usama Taya, Shamardan Ezzeldin S. Bazeed, Zaki F. Aref, Mennatallah Ali Abdelrhman Sayed, Hanaa Nafady-Hego, Aida A. Abdelmaksoud

**Affiliations:** 1 Department of Chest Diseases and Tuberculosis, Faculty of Medicine, South Valley-University, Qena, Egypt; 2 Department of Clinical Pathology, Faculty of Medicine, Assiut University, Assiut, Egypt; 3 Department of Clinical and Chemical Pathology, Faculty of Medicine, South Valley University, Qena, Egypt; 4 Department of Medical Biochemistry, Faculty of Medicine, South Valley University, Qena, 83523 Egypt; 5 ENT Department, Faculty of Medicine, South Valley University, Qena, Egypt; 6 Tropical Medicine and Gastroenterology Department, Faculty of Medicine, South Valley University, Qena, Egypt; 7 King Salman International University, Sharm El-Sheikh, Egypt; 8 Department of Microbiology and Immunology, Faculty of Medicine, Assiut University, Assiut, Egypt

**Keywords:** Biochemistry, Microbiology, Molecular biology, Molecular medicine

## Abstract

Evidence on the efficacy of adding macrolides (azithromycin or clarithromycin) to the treatment regimen for COVID-19 is limited. We testify whether adding azithromycin or clarithromycin to a standard of care regimen was superior to standard of supportive care alone in patients with mild COVID-19.This randomized trial included three groups of patients with COVID-19. The azithromycin group included, 107 patients who received azithromycin 500 mg/24 h for 7 days, the clarithromycin group included 99 patients who received clarithromycin 500 /12 h for 7 days, and the control group included 99 patients who received standard care only. All three groups received only symptomatic treatment for control of fever and cough .Clinical and biochemical evaluations of the study participants including assessment of the symptoms duration, real-time reverse transcription-polymerase chain reaction (rRT-PCR), C-reactive protein (CRP), serum ferritin, D-dimer, complete blood count (CBC), in addition to non-contrast chest computed tomography (CT), were performed. The overall results revealed significant early improvement of symptoms (fever, dyspnea and cough) in patients treated with either azithromycin or clarithromycin compared to control group, also there was significant early conversion of SARS-CoV-2 PCR to negative in patients treated with either azithromycin or clarithromycin compared to control group (*p* < 0.05 for all).There was no significant difference in time to improvement of fever, cough, dyspnea, anosmia, gastrointestinal tract "GIT" symptoms and time to PCR negative conversion between patients treated with azithromycin compared to patients treated with clarithromycin (*p* > 0.05 for all). Follow up chest CT done after 2 weeks of start of treatment showed significant improvement in patients treated with either azithromycin or clarithromycin compared to control group (*p* < 0.05 for all).Adding Clarithromycin or azithromycin to the therapeutic protocols for COVID-19 could be beneficial for early control of fever and early PCR negative conversion in Mild COVID-19.

*Trial registration*: (NCT04622891) www.ClinicalTrials.gov retrospectively registered (November 10, 2020).

## Introduction

SARS-CoV-2 infection is major global health emergency with many countries still experiencing an increase in cases and related fatalities^1,2^.

As of December 30, 2020, Egypt has reported 135,333 cases of COVID-19 and 7,574 deaths. Most cases were asymptomatic or had mild to moderate symptoms as dyspnea, fever, dry cough, sore throat, or malaise^3–6^. Mild cases pass unnoticed but they can transmit the infection and increase the disease burden. Therapeutic strategies vary between using newly developed medications and repurposing existing medications for COVID-19. Drugs as remdesivir, lopinavir/ritonavir; (anti HIV drugs), chloroquine and hydroxychloroquine (antimalarial drugs) were recommended in COVID-19 treatment due to their antiviral activity^4–6^. Other drugs still under trial as tocilizumab and sarilumab can attenuate COVID-19 and associated cytokine storm by interfering with interleukin-6^7,8^, also the mesenchymal stem cell therapy described earlier^9^. The therapeutic use of convalescent plasma therapy in critically ill patients with COVID-19 has recently emerged. Isolating the SARS-CoV-2 disease-associated antibodies from the population of recovered patients would be considerably more advantageous if done in accordance with the disease's regional distribution. For the treatment of SARS-CoV-2 patients, raised antibodies should be manufactured on a large scale. These antibodies could provide an immediate strategy for SARS-CoV-2 treatment while the alternative, more time-consuming process of developing vaccines and new medicines continues^10^.

Azithromycin is a widely available drug that might decrease viral load when added to hydroxychloroquine in patients with non-severe COVID-19^11,12^. Furthermore, preclinical studies have reported immunomodulatory effects of azithromycin and other macrolides, which could control the intense inflammatory responses that might cause progression to organ failure^4–7^.

Macrolides are bacteriostatic antibiotics, which are widely used in clinical practice against respiratory tract pathogens as gram-positive and atypical bacterial have been shown to have immunomodulatory and anti-inflammatory effects^13–15^. Macrolides have a glycosylic bond connects amino sugar and/or neutral sugar moieties to form a macro-lactone ring of variable size (12–22 carbon atoms). The only 15-membered ring is azithromycin (AZI), a macrolide derivative known as an azalide. (CLA) is 14-membered macrolide, has largely replaced erythromycin (ERY) in clinical practice^16,17^. The anti-inflammatory potential of macrolides to reduce the production of matrix metalloproteinases (MMPs), tumor necrosis factor-alpha (TNF−), interleukin (IL)-6, and IL-8 has been reported in critical cases^18^.

The antibiotic azithromycin suppressed the increase in mucin and TNF-α levels in respiratory epithelial cells from several human patients. It also helps to repair damaged tissues caused by inflammation because of its connection to the T-helper (TH) phenotype. Clarithromycin could regulate viral respiratory tract inflammatory infections by lowering sialic acid α 2,6-galactose sialyloligosaccharides (SA2,6Gal), partially inhibiting NF-κB, and raising endosome pH in respiratory epithelial cells^19–21^. The anti inflammatory mechanism of clarithromycin through inhibiting the activation of NF-kB in cell nuclei and transcription reduction make it a good candidate drug in COVID-19 setting as most of the disease pathology is due to inflammation^22^. Together with the suppressing effect of clarithromycin on SAα 2,6Gal, a receptor for human influenza located on human tracheae surface mucosa, and prevents viral invasion, will lead to protein synthesis inhibition, translocation of aminoacyl transfer-RNA, and preventing peptide chain elongation. The same scenario can happen in SARS-Cov-2 viral infection^23^.

To date, evidence on the efficacy and safety of adding azithromycin or clarithromycin to the treatment regimen for mild/ moderate COVID-19 is limited by low-quality studies^10,11,14^. The main objective of the current research is to address the effectiveness of these drugs in improving the clinical status and fasten PCR negative conversion in patients with mild COVID-19.

## Patients and methods

### Study design and participants

The current study is a clinical trial conducted at Qena Governorate, Egypt, during the period from May 2020, to September 2020. An institutional ethical committee approval, Faculty of Medicine, South Valley University, Qena, Egypt, was taken, Ethical approval code: SVU202(SVU-CHT019-420860). A written informed consent was taken from all the patients participate in this study and the procedures were in accordance to Helsinki guidelines for human research**.**

**Figure 1:**
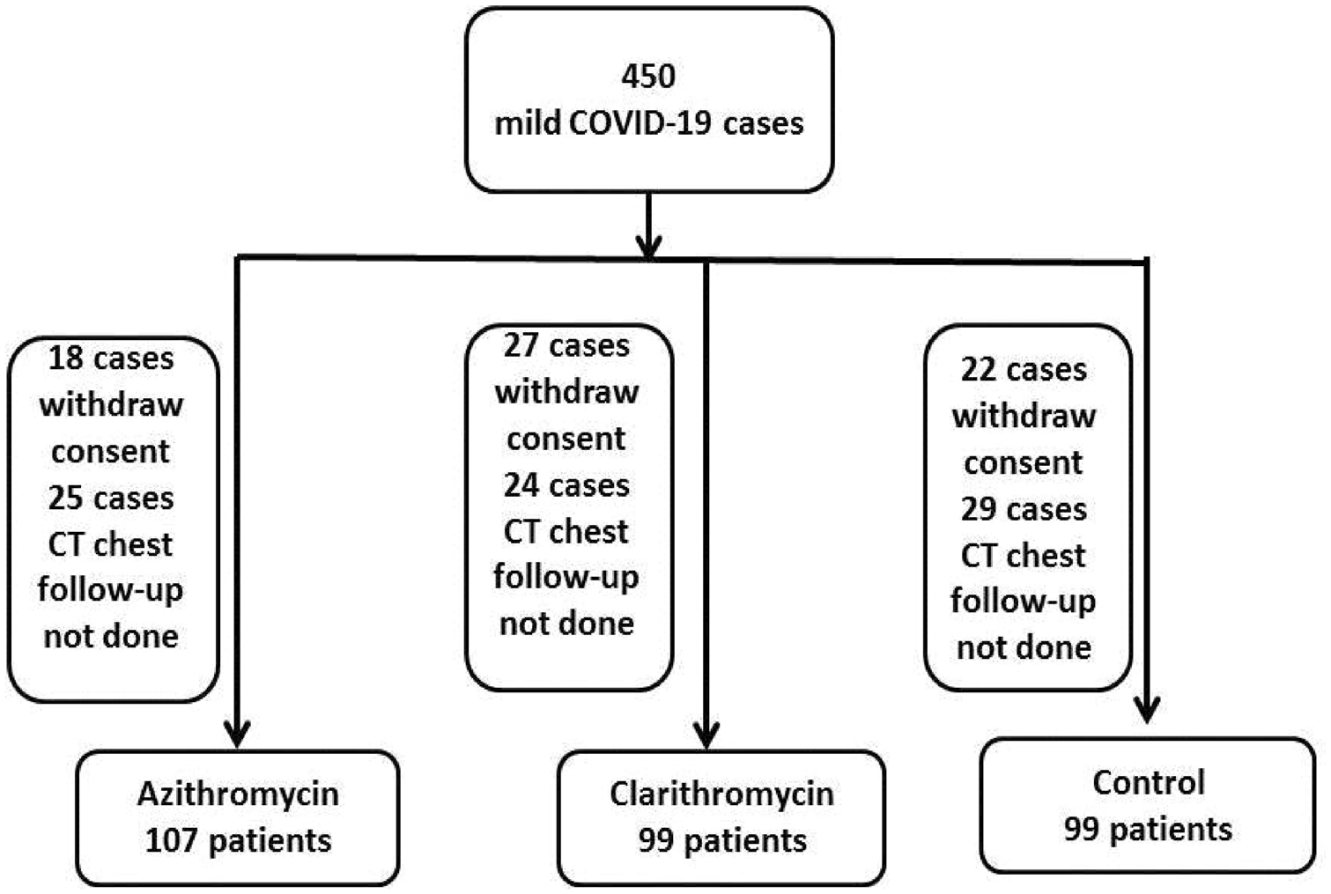
Algorithm of the study design and participants.

Our study included 305 confirmed cases of mild COVID-19 according to WHO classification of clinical cases and all of them received the standard care regimen. They have active COVID-19, Oxygen saturation > 90% on room air and imaging shows less than 25% infiltrates on CT chest. They were randomly assigned to one of three study groups. The azithromycin group included, 107 patients who received oral azithromycin 500 mg/24 h for 7 days, the clarithromycin group included 99 patients who received oral clarithromycin 500 /12 h for 7 days, and the control group included 99 patients who received standard care only**.** All three groups received standard care regimen to control fever (paracetamol) and cough. The three included groups of patients were matched regarding to age, sex, and body mass index (BMI), they were non-smokers and not have any co-morbidities. Patients were followed in intermediate care facility for quarantine of mild COVID-19 cases. The study flow chart done in intermediate care facility for quarantine of mild COVID-19 cases was presented in (Fig. 1). Not all included patients at the start of the study were reported in this data because they did not follow up with CT or consent withdraws either because they were recovered or due to limited funding sources.

All study participants underwent full clinical evaluation including duration of fever, cough, dyspnea, anosmia or gastrointestinal tract "GIT" symptoms, daily evaluation of symptoms was done, fever chart was plotted 3 times daily measurement, patient was considered a febrile if body temprature < 37.2 C for two consecutive days. During the study period, an electroencephalogram (ECG) was performed on a regular basis to detect any potential cardiac arrhythmias caused by macrolides therapy, especially prolonged QT interval.

### Hematological, biochemical and molecular measurements, and imaging

A. Prior to therapy, 6 mls of venous blood were collected from each patient and divided into three parts: (2 mls were evacuated into EDTA tubes for CBCs; 2 mls were evacuated into serum gel separator tubes and allowed to clotted at 37 0C for 30 min until centrifugation at 3500 rpm for 10 min, and the separated sera were used for C-reactive protein (CRP) and ferritin assays, while the remaining 2 mls were calibrated to be evacuated into citrate tubes, and the separated citrated plasma was used for D-dimmer assays after centrifugation) as follows:
1. CBC with differential: automatic cell counter, cell dyne-Ruby (Abbott Diagnostics, Santa Clara, California, USA).
2. D-dimer is measured using a CS-1600 digital blood coagulation analyzer from Japan. Spectrum, Egyptian company of biotechnology, Cairo, Egypt, catalogue N.585002, provided the assay kit. The assay is focused on reinforced immunoturbidimetry, in which monoclonal anti-D-dimer antibodies in the reagent react with D-dimer antigen in the sample to form antigen/antibody complexes that increase the turbidity of work solution. Reference range: up to 0.5 ng/dL.
3. CRP (C-reactive protein): the semi-quantitative latex agglutination test was used to conduct the assays (AVITEX CRP kits; Catalog No. OD023; supplied by Omega Diagnostics, UK). Reference range: < 6 mg/dL.
4. Serum ferritin assays were carried out using commercially available ELISA assay kits (provided by BIOCHECK, INC, 323 VNTAGE Park Dr. Foster City, CA94404-catalog number: BC-1025) and a thermo science ELISA multiskan EX microplate photometer (STAT FAX-2100, USA) according to the manufacturer's protocol. Reference range: 10–232 ng/dL.
B. PCR analysis was carried on aliquots of Universal Transport Medium (UTM) that were used to collect nasopharyngeal swabs (Huachenyang Technology, China). On a Rotor-Gene Q (QIAGEN Hilden, Germany) and QIA symphony platform (QIAGEN, USA), aliquots were extracted and tested with real-time reverse transcription PCR (RT-qPCR) using the QIAamp DSP Virus Spin Kit (QIAGEN Hilden, Germany)^24,25^. The PCR was repeated on the fifth day and every 48 h thereafter till two consecutive negative PCR results were obtained.
C. Non-contrast chest computed tomography (CT) which was repeated 2 weeks after start of treatment. Improvement in CT chest was defined as complete resolution of infiltrates in follow up CT chest done after 15 days from start of treatment.

### Randomization and masking

Patients were randomly assigned (1:1:1) either to; azithromycin plus standard of care, clarithromycin plus standard of care or standard of care alone. Randomization in blocks of variable size (4, 6, and 8) was performed in an electronic case report form system and stratified by age (≥ 50 years vs. < 50 years), CRP, ferritin and d-dimer. Allocation was done by a web-based, automated randomization system. Patients and data interpreter were blinded to study drug assignment, while investigators, and health-care providers were not masked to study drug assignment.

### Statistical analysis

Statistical package of social sciences (SPSS), version (21) (SPSS: An IBM Company, version 21.0, IBM Corporation, Armonk, NY, USA) was used for data analysis. Quantitative data presented as mean ± standard deviation (SD) and qualitative data presented in the form of frequency and percentage. Independent Sample T-test, ANOVA or Chi-square test was used to determine the statistically significant variances among the study groups. Pearson’s correlation was used to compare the studied parameters. P ≤ 0.05 reflected significance.

### Ethical approval

 An institutional ethical committee approval, Faculty of Medicine, South Valley University, Qena, Egypt, was taken, Ethical approval code: SVU202(SVU-CHT019-420860). Clinical trial registration no. is (NCT04622891). First posted November 10, 2020.

### Informed consent

 A written informed consent was taken from all the patients participate in this study and the procedures were in accordance to Helsinki guidelines for human research.

## Results

### Baseline demographic, clinical, laboratory and imaging data of the study groups

The current study included 305 confirmed cases of mild COVID-19 who were allocated into three groups. The azithromycin group included, 107 patients, mean age 45.8 ± 18 years, 73 male and 34 female, the clarithromycin group included 99 patients mean age 46.1 ± 19 years, 68 males and 31 female, the control group included 99 patients, with mean age 41.1 ± 18 years, 73 male and 28 female with non-significant differences between the study groups regarding to the age and sex, indicating matching, p > 0.05 for all (Table 1).

**Table 1:** Baseline characteristics of the study groups.

Variables	Azithromycin Group N = 107	Clarithromycin Group N = 99	Control Group N = 99	P value
Age (mean ± SD)	45.8 ± 18	46.1 ± 19	41.1 ± 18	0.098
Sex : N (%)				
Males	73(68.2%)	68(68.6%)	73(73.7%)	0.638
Females	34 (31.8%)	31 (31.4%)	26 (26.3%)	
Fever N (%)	107 (100%)	99 (100%)	99 (100%)	0.135
Cough N (%)	107 (100%)	96 (97%)	96 (97%)	0.154
Dyspnea N (%)	78 (73%)	86 (87%)	85 (86%)	0.056
Anosmia N (%)	21 (19.6%)	23 (23.2%)	23 (23.2%)	0.768
GIT symptoms N (%)	32 (30%)	40 (40%)	33 (33%)	0274
CRP (mean ± SD, mg/dl)	16.3 ± 15.8	20.6 ± 19.4	18.5 ± 18.1	0.216
D-dimer (mean ± SD, ng/mL)	191 ± 291	182 ± 260	237 ± 285	0.334
Neutrophil/lymphocyte ratio (mean ± SD)	3.2 ± 0.9	3.1 ± 1.2	3 ± 1.2	0.598
Ferritin (mean ± SD, ng/mL)	715 ± 417	790 ± 507	690 ± 477	0.292
CT Chest				
< 5% infiltrates	0	3 (3%)	2 (2%)	0.171
6–25% infiltrates	107 (100%)	96 (97%)	97 (98%)	

As regards the baseline clinical and laboratory data, there were no significant differences in the frequency percentage of fever, cough, dyspnea and anosmia, or the mean ± SD of the baseline CRP, serum ferritin, nutrophil/lymphocyte ratio and D-dimer , or the extent of CT chest infiltrates between azithromycin, clarithromycin or control groups, p > 0.05 for all (Table 1).

**Table 2:** Duration of symptom improvement and PCR conversion to negative in the study groups among patients with mild COVID-19.

Variables	Azithromycin Group N = 107	Clarithromycin Group N = 99	Control Group N = 99	P1 value (1,2)	P2 value (1,3)	P3 value (2,3)	P value ANOVA
Time to PCR –ve ( Mean ± SD, days)	(8.7 ± 2.8)	(8.3 ± 2.6)	(13.2 ± 4.2)	0.351	< 0.0001	< 0.0001	< 0.0001
Fever days ( Mean ± SD)	(5.2 ± 2.3)	(4.9 ± 1.5)	(12.9 ± 2.2)	0.353	< 0.0001	< 0.0001	< 0.000
Cough days (Mean ± SD)	(5.4 ± 2.7)	(5.1 ± 2)	(12.9 ± 2.2)	0.481	< 0.0001	< 0.0001	< 0.0001
Dyspnea days ( Mean ± SD)	(4.6 ± 3.3)	(4.7 ± 2.9)	(9.3 ± 2.7)	0.726	< 0.0001	< 0.0001	< 0.0001
Anosmia days (Mean ± SD)	(0.48 ± 0.9)	(1.2 ± 3)	(0.9 ± 2.3)	0.024	0.208	0.323	0.076
GIT symptoms days (Mean ± SD)	(0.9 ± 1.7)	(1.5 ± 2.4)	(1.2 ± 2)	0.046	0.406	0.250	0.134
CT chest follow-up							
Improved	70 (65%)	76 (77%)	53 (54%)				0.0001

P1 = Azithromycin treated group versus clarithromycin treated group.

P2 = Azithromycin treated group versus control group.

P3 = clarithromycin treated group versus control group.

### Therapeutic efficacies of macrolids in patients with mild COVID-19 compared to standard supportive therapy

There was significant shorter duration of fever (days), in patients treated with either azithromycin (5.2 ± 2.3) or clarithromycin (4.9 ± 1.5) compared to control group (12.9 ± 2.2), P < 0.000. Duration of cough also showed significant fewer days for improvment in patients treated with either azithromycin (5.4 ± 2.7) or clarithromycin (5.1 ± 2) compared to control group (12.9 ± 2.2) P < 0.0001, in addition, dyspnea duration (days) showed significant early improvement in patients treated with either azithromycin (4.6 ± 3.3) or clarithromycin (4.7 ± 2.9) compared to control group (9.3 ± 2.7), P < 0.0001 (Table 2). There was significant early conversion (days) of SARS-CoV-2 PCR to negative in patients treated with either azithromycin (8.7 ± 2.8) or clarithromycin (8.3 ± 2.6) compared to control group (13.2 ± 4.2), P < 0.0001 (Table 2).

There was no significant difference in time to improvement of fever (P = 0.351), cough (P = 0.481), dyspnea (P = 0.726), and time to PCR negative conversion (P = 0.351) between patients treated with azithromycin compared to patients treated with clarithromycin (Table 2).

Follow up chest CT chest done 2 weeks after the start of treatment showed significant improvement in patients treated with either azithromycin 70 (65%) or clarithromycin 76 (77%) compared to control group 53 (54%) P < 0.0001, (Table 2).

In terms of the survival analysis, our study protocol stated that the follow-up period would be 7 days only, and none of the patients would die during that time. Furthermore, none of the patients who were included in the study complained of arrhythmias or chest pain.

## Discussion

COVID-19 care presented significant problems to physicians and specialists, in part due to the disease's novelty and the severe symptoms it causes. Vaccination, despite its recent introduction, is still only used in a limited number of situations. Identifying safe and effective therapeutic options for the treatment of such a highly contagious disease is crucial in this scenario^26^. Antiviral therapies or neutralizing monoclonal antibodies should be used to treat the causal virus as soon as possible; however, treating the underlying inflammatory disease may not be beneficial. The antiviral effect of macrolids was originally discovered in individuals with widespread pan bronchitis who were given a modest dose of erythromycin and saw a reduction in inflammation in the upper and lower respiratory tract^27^. Clarithromycin's efficacy against viral infections such as rhinovirus (RV), respiratory syncytial virus (RSV), and influenza virus has been studied previously^28–31^.

In this trial, two groups of patients were randomly assigned to receive either azithromycin or clarithromycin in addition to standard care, and were compared to a third group that only got standard care. The average mean of age was comparable among the 3 study groups (azithromycin was 45.8 years, clarithromycin was 46.1 years, and control group was 41.1 years respectively, P > 0.05). Before starting the therapy regimen, the clinical and laboratory investigations of the patients do not differ. Fever, dyspnea, and cough improved significantly, and SARS-CoV-2 PCR was converted to negative in both azithromycin and clarithromycin groups earlier than in the control group. The benefit–risk profile of macrolides in COVID-19 patients is currently being discovered. According to clinicaltrials.gov, there are now 20 ongoing research trials using azithromycin, either alone or in combination with other drugs, in COVID-19^32^. Azithromycin pretreatment of COVID-19 patients can minimize LPS-induced lung bioluminescence and airway cell infiltration, lower the amount of proinflammatory cytokines in bronchoalveolar lavage, and inhibit NF-kβ nuclear translocation, according to a study by Stellari et al.^33^. In the other hand, a total of 1438 hospitalized patients with COVID-19 were included in one trial. 735 (51.1%) were given hydroxychloroquine with azithromycin, 271 (18.8%) were given hydroxychloroquine alone, 211 (14.7%) were given azithromycin alone, and 221 (15.4%) were given other medications. In-hospital mortality did not differ significantly when statistical analyses were adjusted^34^. It's important to note that their patients weren't in the same circumstances as ours, who were mild cases for which azithromycin or clarithromycin would still have a chance to suppress the immune response and lower cytokine expression before severe inflammation and problems developed. They also failed to account for the time required for PCR negative conversion. In agreement, Gautret et al. reported that in six patients, combining azithromycin with hydroxychloroquine resulted in statistically higher viral clearance (6/6, 100%) compared to hydroxychloroquine monotherapy (8/14, 57%)^35^. In contrast, a report that comprised randomized trials comparing macrolides—as monotherapy or in combination with other medications—to placebo or no treatment in patients with COVID-19 indicated that macrolides had no benefit in the management of COVID-19 patients compared to standard of care. All of the evidence was inconclusive, thus a larger trial was advised^36^. Cavalcanti et al. found that hydroxychloroquine, either alone or in combination with azithromycin, did not improve clinical status at 15 days as compared to standard therapy in patients hospitalized with mild-to-moderate COVID-19^37^.

The use of a chest CT scan in the early diagnosis of COVID-19 patients is critical^35^. As time passes as the disease progresses, the radiological results tend to become more confluent and bilateral. Dense consolidations with ground glass opacities (GGOs) are an example of this^36^. There was a substantial reduction in lung infiltrates in patients treated with azithromycin or clarithromycin compared to the control group when it came to the effect of macrolides therapy on radiological findings of the included patients. In confirmation of azithromycin's importance in treating COVID-19, a survey of 6200 physicians in 30 countries found that azithromycin was the second most regularly recommended antibiotic for COVID-19 treatment^37^. Based on our results we concluded that, azithromycin or clarithromycin when used in mild COVID-19 cases are safe and effective adjunctive therapy for alleviating the early symptoms, viral shedding that can reduce viral transmission.

## Conclusion

The current study provides evidence for the use of macrolids (clarithromycin or azithromycin) in mild COVID-19 treatment protocols, which could be effective for early fever management and PCR negative conversion. More research into macrolids' safety and efficacy, as well as their use in conjunction with other regularly used therapy modalities, is needed.

## Study limitations

The study's main limitations were the small sample size, single-center analysis, and absence of long-term comparative follow-up of the included patients to determine any late complications, protection duration, and the probability of re-infection after complete COVID-19 cure.
